# 2355. Prison as a Driver of Recent Transmissions of Multidrug-Resistant Tuberculosis in Callao, Peru

**DOI:** 10.1093/ofid/ofac492.162

**Published:** 2022-12-15

**Authors:** Christian Utpatel, Milagros Zavaleta, Daniel E Rojas-Bolivar, Andreas Mulhbach, Janet Picoy, Walter Jose J Portugal, Ana Esteve-Sole, Laia Alsina, Paolo Miotto, Daniella Bartholomeu, Jorge Sanchez, Jorge O Alarcon, Stefan Niemann, Moises A Huaman

**Affiliations:** Molecular and Experimental Mycobacteriology, Research Center Borstel, Borstel, Germany, Borstel, Schleswig-Holstein, Germany; Centro de Investigaciones Tecnológicas, Biomedicas y Medioambientales, Callao, Peru, Callao, Callao, Peru; Centro de Investigaciones Tecnológicas, Biomedicas y Medioambientales, Callao, Peru, Callao, Callao, Peru; Centro de Investigaciones Tecnológicas, Biomedicas y Medioambientales, Callao, Peru, Callao, Callao, Peru; Direccion Regional de Salud del Callao, Callao, Peru, Callao, Callao, Peru; Direccion Regional de Salud del Callao, Callao, Peru, Callao, Callao, Peru; Allergy and Clinical Immunology Department, Hospital Sant Joan de Déu Institut de Recerca Sant Joan de Déu, University of Barcelona, Barcelona, Spain, Barcelona, Catalonia, Spain; Allergy and Clinical Immunology Department, Hospital Sant Joan de Déu Institut de Recerca Sant Joan de Déu, University of Barcelona, Barcelona, Spain, Barcelona, Catalonia, Spain; IRCCS San Raffaele Scientific Institute, Milan, Italy, Milan, Lombardia, Italy; Universidade Federal Minas Gerais, Brazil, Belo Horizonte, Minas Gerais, Brazil; Centro de Investigaciones Tecnológicas, Biomedicas y Medioambientales, Callao, Peru, Callao, Callao, Peru; Centro de Investigaciones Tecnológicas, Biomedicas y Medioambientales; Epidemiology Section, Instituto de Medicina Tropical, Universidad Nacional Mayor de San Marcos, Peru, Callao, Callao, Peru; Molecular and Experimental Mycobacteriology, Research Center Borstel; German Center for Infection Research, Partner Site Hamburg-Lübeck-Borstel-Riems, Borstel, Germany, Borstel, Schleswig-Holstein, Germany; Division of Infectious Diseases, University of Cincinnati, Cincinnati, USA, Cincinnati, OH

## Abstract

**Background:**

Peru has one of the highest rates of multidrug-resistant tuberculosis (MDR-TB) in the Latin America region, with Callao being one of the hot spots. We sought to identify resistance patterns and key drivers of recent MDR-TB transmission in Callao, Peru.

**Methods:**

Cross-sectional study including clinical specimens identified as MDR-TB strains in Callao, Peru between April 2017 and December 2019. DNA was extracted for whole genome sequencing and data used for phylogenetic classification, clustering, and resistance causing mutation analyses. Recent transmission was defined based on an isolate-to-isolate distance of ≤5 (D5) single nucleotide polymorphisms (SNPs). We used logistic regression models to analyze the relationship between MDR-TB clustering and epidemiologic factors including age, sex, history of diabetes mellitus, HIV infection, illicit drug use, known TB contact, prior TB disease, and imprisonment.

**Results:**

171 unique MDR-TB strains were included; 93% were assigned to lineage 4 and 7% to lineage 2. The most prevalent sublineage was 4.3.3 LAM (57%), followed by 4.3.4.2 LAM (10%) and 4.1.2.1 Haarlem (9%). In the dominant 4.3.3 LAM sublineage, concomitant resistance was common, including resistance mutations to pyrazinamide (92%), ethambutol (22%), ethionamide (23%), and quinolones (10%); 4 isolates harbored bedaquiline resistance mutations. Seventy-four percent of 4.3.3 LAM isolates were D5-clustered, with 53 (30%) isolates within a single dominant cluster. Male sex (odds ratio [OR], 3.6; 95% confidence interval [CI], 1.5 – 8.9), drug use (OR, 4.9; 95% CI, 1.9 – 12.7), and current or prior history of imprisonment (OR, 12; 95% CI, 3.3 – 43.5) were associated with the dominant D5 cluster in unadjusted analyses. History of imprisonment remained independently associated with the dominant D5 cluster in multivariate analyses (adjusted OR, 8.9; 95% CI, 1.6 – 50.6). History of imprisonment was also independently associated with being part of any D5 clusters in multivariate analyses (adjusted OR, 4.8; 95% CI, 1.2 – 20.3).

**Conclusion:**

History of imprisonment was linked to current MDR-TB transmissions, indicating an important role of prisons in driving the MDR-TB epidemic.

**Disclosures:**

**Moises A. Huaman, MD, MSc**, Gilead: Grant/Research Support|Insmed: Grant/Research Support.

